# Longitudinal Clinical Outcomes of Tissue‐Level Dental Implants in a Periodontal Practice: A Retrospective Study

**DOI:** 10.1111/cid.70132

**Published:** 2026-02-27

**Authors:** Hongmin Lai, Ming‐Tsuen Lin, Ting‐Yu Lin, Sam Li‐Sheng Chen, Tony Hsiu‐Hsi Chen, Amy Ming‐Fang Yen

**Affiliations:** ^1^ ATEK Elite Dental Clinic Taipei Taiwan; ^2^ Dental Department Taipei Medical University Shuang‐Ho Hospital New Taipei City Taiwan; ^3^ Lin Yeh Dental Clinic Taipei Taiwan; ^4^ Institute of Health Data Analytics and Statistics, College of Public Health, National Taiwan University Taipei Taiwan; ^5^ School of Oral Hygiene College of Oral Medicine, Taipei Medical University Taipei Taiwan

**Keywords:** dental implants, maintenance, peri‐implantitis, periodontitis, retrospective studies, risk factors, survival

## Abstract

**Objectives:**

This study aimed to evaluate the 28‐year cumulative success and survival rates of tissue‐level implants placed by a periodontist in Taiwan.

**Materials and Methods:**

In this retrospective cohort study, 685 tissue‐level implants placed in 199 patients between 1996 and 2024 were analyzed. A success was defined as the absence of probing depth (PD) ≥ 5 mm nor implant loss—while survival referred to the presence and clinical function of the implant. Kaplan–Meier analysis was used to estimate cumulative rates at both implant and patient levels. Potential risk factors, including patient demographics, smoking status, implant characteristics, periodontal status, and frequency of maintenance visits, were examined using Cox proportional hazards regression models.

**Results:**

At 28 years, the cumulative success rates were 71.00% at the implant level and 38.02% at the patient level, while the survival rates were 92.58% and 81.38%, respectively. Multivariable regression analysis identified male and severe periodontitis as significant predictors of peri‐implant disease or implant loss. In contrast, more frequent maintenance visits were protective. Treatment outcomes for peri‐implantitis were variable and difficult to predict.

**Conclusions:**

Tissue‐level implants demonstrated favorable long‐term clinical performance in a Taiwanese population, with success and survival rates comparable to those reported in Western cohorts. These findings underscore the importance of periodontal health and regular maintenance care in sustaining implant longevity.

## 1 Introduction

Tissue‐level dental implants are among the most widely used systems globally. The performance of tissue‐level implants has been recognized as exceptional, with reported cumulative success and survival rates exceeding 90% in several studies [1, 2]. Nevertheless, these rates tend to decline with extended follow‐up durations [3]. Several risk indicators for complications and implant loss have been identified, including placement in the maxillary arch [1, 4], pre‐existing periodontal disease [1, 5, 6, 7], smoking [1, 8, 9], short implant length [1, 10, 11], hydroxyapatite (HA) surface coatings [10], and restorations provided by general practitioners [2].

Long‐term success and survival rates of tissue‐level implants have been well documented in Western countries, including Switzerland [12], Italy [11, 13], Belgium [5], France [8], Canada [9], and across multi‐national cohorts [14]. However, to our knowledge, there is a lack of comparable studies with follow‐up durations exceeding 20 years in the Asia‐Pacific region.

Pocket formation is one of the most prevalent biological complications associated with dental implants and is considered a defining characteristic of peri‐implantitis [15, 16, 17, 18, 19, 20]. Probing depth measurement remains the simplest and most direct clinical method for diagnosing peri‐implant complications. However, treatment outcomes for peri‐implant pockets remain unpredictable [16, 18, 21, 22, 23]. Achieving a pocket‐free status is therefore regarded as a crucial indicator of long‐term implant stability.

Despite this, most diagnostic classifications of peri‐implantitis rely on a triad of indicators: pocket formation, bleeding or suppuration on probing (BOP), and radiographic evidence of bone loss [15, 17, 22, 24]. This multi‐criterion approach raises the diagnostic threshold, reducing sensitivity and potentially leading to false‐negative assessments. Increasing the threshold or the number of criteria significantly decreases the prevalence of peri‐implantitis [25, 26].

Additionally, the utility of conventional radiographs is difficult to detect bone loss on the buccal and lingual aspects. Even the interdental images of bone loss can be obscured by bone density, further undermining radiographic accuracy. While BOP is known to have low diagnostic sensitivity but high specificity for periodontal tissue breakdown [27, 28], this limitation can also extend to peri‐implant evaluations [29]. Notably, pocket formation alone may signify compromised osseointegration and thus serve as a practical and early indicator of peri‐implant diseases, even before radiographic evidence of bone loss becomes apparent. Using pocket formation as a sole diagnostic criterion may therefore enhance sensitivity and enable earlier detection, while also offering simplicity and clinical practicality.

The present study aimed to evaluate the 28‐year cumulative success and survival rates of tissue‐level implants placed by a periodontist in Taiwan.

## 2 Materials and Methods

### 2.1 Study Design and Population

This retrospective cohort study included patients treated at the private periodontal practice of the author (H.L.), a board‐certified periodontist, between August 1996 and May 2024. All clinical examinations, periodontal therapy, implant surgeries, and maintenance procedures were performed by the author because Taiwan has no dental hygienist system. The inclusion criteria were: (1) Taiwanese adults aged ≧ 20 years; and (2) patients who received Straumann tissue‐level solid screw‐type implants. Implants inserted prior to 2000 featured a titanium plasma‐sprayed (TPS) surface, while those placed subsequently utilized a sand‐blasted, large‐grit, acid‐etched (SLA) surface. The exclusion criteria were: (1) patients with poorly controlled diabetes (HbA1c > 8.0%) [30]; (2) heavy smokers (≥ 20 cigarettes/day); (3) non‐Taiwanese individuals; and (4) patients receiving implant systems other than the study device.

### 2.2 Surgical Procedures and Maintenance

Before implant placement, all patients received periodontal therapy to restore oral health, particularly around teeth adjacent to the implant sites. Preoperative assessments were conducted using cone beam computed tomography (CBCT), with the exception of a few early cases where medical‐grade spiral CT was employed. In cases where buccal dehiscence of the alveolar plate was observed, bone grafting—with or without a barrier membrane—was performed. Autogenous bone alone was used for dehiscences ≤ 3 mm; for larger defects, guided bone regeneration (GBR) was performed using allografts and predominantly resorbable membranes. All implants were placed using a non‐submerged protocol. Antibiotics were administered postoperatively primarily in GBR, sinus augmentation cases, or during first half cases when experience was still cumulating. Chlorhexidine rinse was not prescribed postoperatively. Sutures were removed 1 week after surgery. Postoperative care was arranged weekly for 2 weeks then monthly till the case was referred to the restorative dentist. Postoperative care includes cleaning of the non‐submerged parts of the fixtures and the adjacent teeth. Patients were instructed to clean fixture heads with moistened cotton balls for the first 2 weeks then followed by gentle brushing.

The healing period between fixture placement and abutment connection varied by case type: for early and GBR cases, healing spanned 3–6 months, while for later standard cases, the interval was shortened to 6–8 weeks [11]. Abutment connections and prosthetic procedures were carried out by restorative dentists. Patients were scheduled for maintenance visits every three months to one year, depending on their periodontal and peri‐implant status. Each visit, lasting approximately one hour, included clinical probing, examination of periodontal and peri‐implant tissues, and thorough debridement of both natural teeth and implants. When probing depths ≥ 5 mm were detected around an implant, deep debridement under local anesthesia was performed by the first author (H. L.). Persistent pockets after 1 month prompted further surgical intervention. Implants exhibiting mobility were classified as failures and were subsequently removed.

This study was approved by the Institutional Review Board (IRB) of Taipei Medical University–Joint Institutional Review Board (TMU‐JIRB; Approval No.: N202206072). The requirement for obtaining informed consent was waived by the IRB because the study was retrospective and involved no more than minimal risk to participants. The study was conducted in accordance with the Declaration of Helsinki and applicable regulatory guidelines for research involving human participants.

### 2.3 Measurements

Modified Plaque Control Record (PCR) of O'Leary et al. [31] was used for patients' oral hygiene. While the original system records biofilm at buccal, lingual, mesial, and distal surfaces, we recorded six surfaces by extending interproximal surfaces to mesiobuccal, mesiolingual, distobuccal, and distolingual surfaces. Patients' best oral hygiene was used. When full‐mouth PCR score was ≦ 10%, it was defined as excellent, 11% to 20% as good, and > 20% as poor.

Baseline demographic data and periodontal conditions were recorded prior to implant placement. Periodontal status of both teeth and implants was evaluated using probing depth (PD) based on a modified version of Roccuzzo's scoring system [6]: S = 1 × PD (5–7 mm) + 2 × PD (≥ 8 mm), calculated per site. Patients were classified as periodontally healthy (PHP, S = 0), moderately compromised (PCP, S ≤ 25), or severely compromised (S > 25). Since some patients already had implants with pockets before our implant placements, these pockets could affect the health of new implants. Therefore, we extended the original four‐site scoring system to conventional six sites per tooth or implant, added a mild category of S = 1–10, and modified moderate category as S = 11–25. Since Roccuzzo's scoring system is based on pockets, a system based on clinical attachment loss (CAL) such as the periodontitis stages of the 2017 World Workshop on the Classification of Periodontal and Peri‐implant Diseases and Conditions (2017 WW) [32] was also considered. There is a borderland between gingivitis and stage I periodontitis, thus gingivitis was incorporated into stage I.

Keratinized mucosa (KM) is pink and immobile, while alveolar mucosa (AM) is red and mobile [33]. AM was determined when the mucosa was rolled at pushing with the side of a periodontal probe or a blunt handle end of a dental instrument.

Postoperative infection was defined as soft tissue abscess or acute inflammation occurring within 2 weeks of implant surgery. Early implant loss was defined as loss before abutment connection, and late implant loss as loss after abutment connection.

A healthy implant was defined as immobile, no BOP, and PD < 5 mm. In this study, peri‐implant disease (PID) was defined as an implant with PD ≥ 5 mm regardless of BOP [15, 16, 17, 34, 35]. The success rate was calculated as 1 minus the rates of PID and loss, while the survival rate as 1 minus the implant loss rate. Maintenance frequency was computed as the number of maintenance appointments divided by the total follow‐up years.

### 2.4 Statistical Analysis

Only Taiwanese patients were included in the final analysis. Data from five Caucasian patients (13 implants) and two sleeping fixtures were excluded. Patient age was recorded at the time of each implant placement. Follow‐up duration was expressed in implant‐years. Kaplan–Meier survival analysis was employed to estimate cumulative success and survival rates. Accounting for clustering effects due to multiple implants per patient, rates were reported at both the implant and patient levels.

Potential risk factors, including sex, age at implantation, maintenance frequency, dental arch, smoking status, implant dimensions (diameter and length), surface type, presence of KM, grafting procedures, sinus lift, baseline periodontal condition of adjacent teeth, full‐mouth Roccuzzo scores, and periodontitis stage scores (I to IV) and grades (A to C) of 2017 WW [32]—were examined using univariate and multivariable Cox proportional hazards models. Outcomes of interest included PID and implant loss. Both crude hazard ratios (cHR) based on univariate analysis and adjusted hazard ratios (aHR) on multivariate analysis with 95% confidence intervals (CI) were reported. A two‐tailed *p*‐value of < 0.05 was considered statistically significant.

## 3 Results

### 3.1 General and Surgical Outcomes

This study analyzed 685 tissue‐level implants placed in 199 Taiwanese patients, comprising 86 males and 113 females, with a mean age of 55.07 years at the time of first implant placement. The mean follow‐up duration was 8.34 implant‐years. A total of 411 surgeries were conducted: 198 surgeries involved the placement of 313 implants in the maxillae, and 213 surgeries involved 372 implants in the mandibles. Due to multiple procedures performed at different sites during some appointments, the total number of surgical appointments amounted to 358.

Antibiotic use was recorded for 129 appointments, while 229 appointments were completed without antibiotics. The overall incidence of postoperative infection was as low as 5.9%. Nine cases (7.0%) occurred in the antibiotic appointments and 12 cases (5.2%) in the non‐antibiotic group. Antibiotic use did not reduce the incidence of postoperative infection (*χ* ^2^ = 0.045).

For the best oral hygiene, 79 patients were excellent, 83 were good, 21 were poor, and 16 patients' data were missing. Overall, most patients could achieve good oral hygiene.

### 3.2 Clustering Effects

A total of 99 implants developed PID and 24 implants were lost. Of these, 8 implants exhibited both conditions, yielding 115 implants classified as having complications. At the patient level, 63 individuals developed PID, and 15 experienced implant loss; 8 patients exhibited both, resulting in 70 patients with complications. For PID, 55 implants (55.0%) were found in 19 of the 63 patients (30.2%). Similarly, 13 lost implants (54.1%) were concentrated among 4 of the 15 patients (26.7%). Of the 24 lost implants, 14 (58.3%) occurred up to 3.8 months postoperatively, before referring for abutment connections. By the definition of this study, they were early loss.

### 3.3 Keratinized Mucosa

As KM can exist on either the buccal or lingual side, its association with PID was assessed at the side level, while its association with implant loss was analyzed at the implant level. Due to incomplete documentation, KM was categorized into three groups: present, absent, and not recorded (Table 1).

**TABLE 1:** Characteristics and the proportions of peri‐implant disease and loss.

	Implant	Peri‐implant disease		Implant loss	
	*n*	*n*	%	*n*	%
Total	685	115	16.79	24	3.50
Sex					
Male	325	70	21.54	19	5.85
Female	360	45	12.50	5	1.39
Age at each implant					
< 50	127	25	19.69	9	7.09
50–59	267	49	18.35	6	2.25
60–69	242	35	14.46	7	2.89
70+	49	6	12.24	2	4.08
Maintenance frequency/y	1.37 ± 0.81	1.35 ± 0.91		0.58 ± 1.04	
Arch					
Maxillary	314	66	21.02	12	3.82
Mandible	371	49	13.21	12	3.23
Smoking					
Yes	32	9	28.13	4	12.50
No	653	106	16.23	20	3.06
Implant diameter (mm)					
3.3	104	21	20.19	8	7.69
4.1	369	61	16.53	9	2.44
4.8	212	33	15.57	7	3.30
Implant length (mm)					
6	6	0	0.00	0	0.00
8	65	15	23.08	11	16.92
10	326	55	16.87	7	2.15
12	258	35	13.57	3	1.16
14	29	10	34.48	3	10.34
16	1	0	0.00	0	0.00
Implant surface					
TPA	45	4	8.89	1	2.22
SLA	640	111	17.34	23	3.59
Bone grafting or sinus lift					
Yes	350	69	19.71	15	4.29
No	335	46	13.73	9	2.69
Buccal mucosa					
Keratinized	349	75	21.49	15	4.30
Non‐keratinized	218	30	13.76	5	2.29
Not recorded	118	10	8.47	4	3.39
Lingual mucosa					
Keratinized	665	108	16.24	24	3.61
Non‐keratinized	20	7	35.00	0	0.00
Adjacent PD^a^					
≤ 3 mm	612	97	15.85	21	3.43
4+ mm	73	18	24.66	3	4.11
Roccuzzo score					
0	356	46	12.92	8	2.25
1–10	299	57	19.06	10	3.34
11–24	25	7	28.00	3	12.00
25+	5	5	100.00	3	60.00
Stage					
1	108	10	9.26	1	0.93
2	41	7	17.07	0	0.00
3	74	12	16.22	1	1.35
4	462	86	18.61	22	4.76
Grade					
A	105	17	16.19	2	1.90
B	219	25	11.42	5	2.28
C	361	73	20.22	17	4.71

^a^ PD, probing depth.

### 3.4 Implant Characteristics and Prosthetic Restorations

Distributions of implant characteristics are summarized in Table 1. A total of 14 clinicians provided prosthetic crowns; one prosthodontist (M‐T. Lin) was responsible for delivering 547 (79.85%) of the 685 restorations.

Among 685 implants, there were six three‐unit implant bridges in four patients; one implant had PID. Two patients had mandibular overdentures on nine implants; one implant had PID.

### 3.5 Cumulative Success and Survival Rates

The distributions of the numbers of implants that had 5‐, 10‐, 15‐, 20‐, and 28‐year follow‐up were 428, 246, 136, 53, and 1, respectively.

**FIGURE 1:**
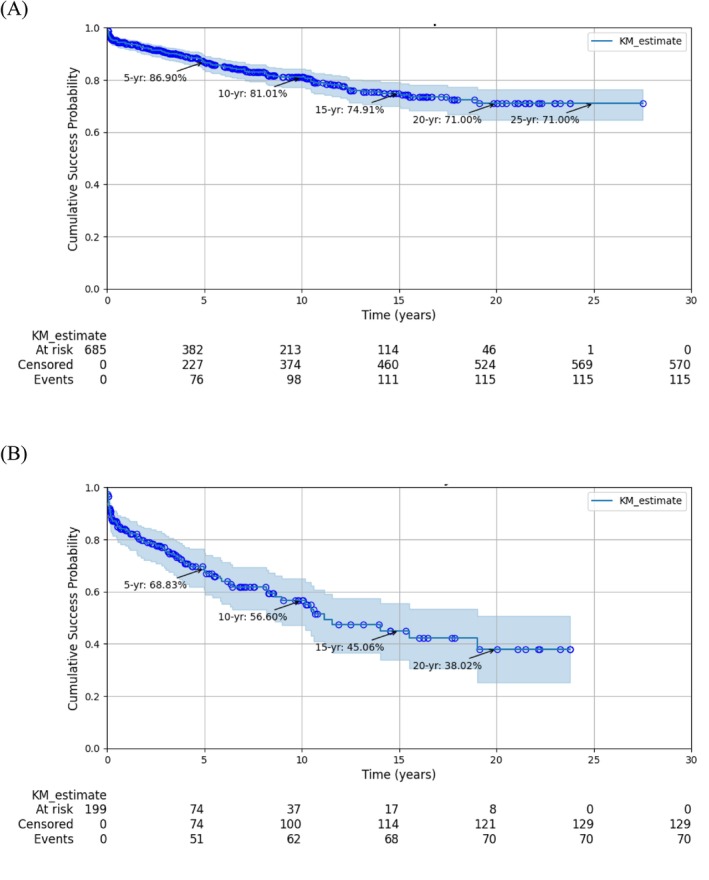
Success rate of tissue‐level implants. (A) Implant level. (B) At subject level.

**FIGURE 2:**
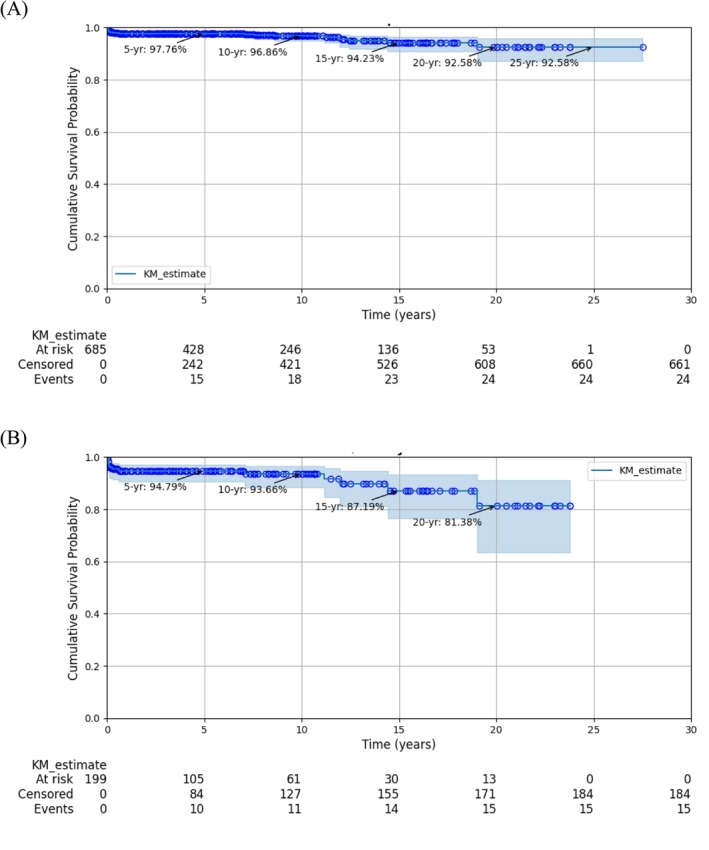
Survival rate of tissue‐level implants. (A) At implant level. (B) At subject level.

The 28‐year cumulative success rate was 71.00% at the implant level (Figure 1A) and 38.02% at the patient level (Figure 1B). The corresponding survival rates were 92.58% (Figure 2A) and 81.38% (Figure 2B), respectively.

### 3.6 Risk Factors for PID and Implant Loss

For PID, univariate Cox proportional hazards regression identified significant risk factors as male, maxillary placement, Roccuzzo's moderate and severe periodontitis, and smoking (marginal risk). Non‐KM was a protective factor. In multivariable analysis, male and Roccuzzo's severe periodontitis remained significant risk factors. Non‐KM remained a protective factor while increasing maintenance frequency became marginally protective (Table 2).

**TABLE 2:** Results of the Cox regression model for risk factors associated with peri‐implant disease.

Variables	Level	cHR	aHR_1_	aHR_2_
Sex	Male	1.86 (1.28, 2.70)	1.82 (1.21, 2.75)	1.71 (1.12, 2.62)
	Female	1.00	1.00	1.00
Age at each implant	Per 10 y increase	1.00 (0.98, 1.02)	0.98 (0.92, 1.04)	0.98 (0.92, 1.04)
Age of the first implant	Per 10 y increase	1.00 (0.98, 1.02)	1.02 (0.96, 1.09)	1.02 (0.96, 1.09)
Maintenance frequency/y		0.80 (0.61, 1.04)	0.72 (0.52, 1.00)	0.68 (0.49, 0.96)
Arch	Maxilla	1.52 (1.05, 2.20)	1.33 (0.86, 2.06)	1.35 (0.87, 2.08)
	Mandible	1.00	1.00	1.00
Smoking	Yes	1.98 (1.00, 3.91)	0.58 (0.22, 1.56)	0.54 (0.20, 1.49)
	No	1.00	1.00	1.00
Implant diameter	Per 1 mm increase	0.87 (0.61, 1.25)	0.86 (0.59, 1.25)	0.86 (0.59, 1.25)
Implant length	Per 1 mm increase	0.99 (0.89, 1.12)	0.88 (0.77, 1.00)	0.88 (0.77, 1.01)
Implant surface	TPA	1.00	1.00	1.00
	SLA	2.45 (0.90, 6.67)	2.39 (0.84, 6.81)	2.29 (0.79, 6.62)
Bone grafting or sinus lift	No	1.00	1.00	1.00
	Yes	1.39 (0.95, 2.01)	1.07 (0.70, 1.63)	1.07 (0.70, 1.63)
Mucosa^a^	Keratinized	1.00	1.00	1.00
	Non‐keratinized	0.47 (0.31, 0.72)	0.54 (0.34, 0.86)	0.52 (0.33, 0.84)
	Not recorded	0.61 (0.32, 1.19)	0.72 (0.34, 1.53)	0.74 (0.34, 1.63)
Adjacent PD	≤ 3 mm	1.00	1.00	1.00
	4+ mm	1.59 (0.96, 2.62)	1.25 (0.71, 2.20)	1.20 (0.68, 2.12)
Roccuzzo score	0	1.00	1.00	1.00
	1–10	1.26 (0.85, 1.86)	1.35 (0.88, 2.07)	1.32 (0.84, 2.07)
	11–24	2.45 (1.10, 5.42)	2.20 (0.95, 5.11)	2.20 (0.92, 5.26)
	25+	6.66 (2.64, 16.80)	4.90 (1.29, 18.69)	4.91 (1.24, 19.43)
Stage	1	1.00		1.00
	2	1.57 (0.60, 4.12)		1.81 (0.61, 5.37)
	3	1.79 (0.77, 4.15)		1.47 (0.52, 4.18)
	4	1.37 (0.71, 2.64)		1.41 (0.56, 3.56)
Grade	A	1.00		1.00
	B	0.68 (0.37, 1.26)		0.57 (0.28, 1.16)
	C	1.05 (0.62, 1.78)		0.81 (0.40, 1.64)

*Note:* Crude values are univariate. aHR_1_ adjusts for sex, age, maintenance, arch, smoking, implant/grafting parameters, keratinized mucosa, adjacent PD, and Roccuzzo score. aHR_2_ further adjusts for periodontitis stage/grade (2017 WW).

Abbreviations: HR, hazard ratio; PD, probing depth.

^a^ Side level.

For implant loss, univariate Cox proportional hazards regression identified male, smoking, and Roccuzzo's moderate and severe periodontitis as significant risk factors, while increasing maintenance frequency and longer implant length were protective, increasing implant age was marginal protective. In the multivariable model, male, Roccuzzo's mild periodontitis and severe periodontitis were significant risk factors. Increasing frequent maintenance and longer implant length remained protective (Table 3).

**TABLE 3:** Results of the Cox regression model for risk factors associated with implant loss.

Variables	Level	cHR	aHR_1_	aHR_2_
Sex	Male	4.39 (1.64, 11.77)	5.55 (1.75, 17.68)	5.43 (1.63, 18.09)
	Female	1.00	1.00	1.00
Age at each implant	Per 10 y increase	0.96 (0.93, 1.00)	1.06 (0.87, 1.28)	1.06 (0.87, 1.28)
Age of the first implant	Per 10 y increase	0.97 (0.93, 1.01)	0.94 (0.77, 1.15)	0.93 (0.76, 1.15)
Maintenance frequency/y		0.16 (0.08, 0.31)	0.11 (0.05, 0.24)	0.10 (0.05, 0.24)
Arch	Maxilla	1.07 (0.48, 2.39)	2.33 (0.85, 6.41)	2.18 (0.75, 6.31)
	Mandible	1.00	1.00	1.00
Smoking	Yes	4.77 (1.62, 14.10)	0.94 (0.15, 5.93)	0.44 (0.05, 3.69)
	No	1.00	1.00	1.00
Implant diameter	Per 1 mm increase	0.57 (0.27, 1.22)	0.61 (0.23, 1.61)	0.67 (0.25, 1.83)
Implant length	Per 1 mm increase	0.73 (0.58, 0.92)	0.48 (0.34, 0.69)	0.52 (0.36, 0.75)
Implant surface	TPA	1.00	1.00	1.00
	SLA	2.18 (0.29, 16.37)	2.01 (0.21, 18.88)	2.42 (0.25, 23.38)
Bone grafting or sinus lift	No	1.00	1.00	1.00
	Yes	1.57 (0.69, 3.58)	1.11 (0.42, 2.94)	0.97 (0.35, 2.66)
Mucosa^a^	Keratinized	1.00	1.00	1.00
	Non‐keratinized	0.42 (0.15, 1.15)	0.94 (0.27, 3.23)	0.93 (0.26, 3.28)
	Not recorded	1.15 (0.37, 3.51)	1.21 (0.31, 4.67)	1.61 (0.39, 6.66)
Adjacent PD	≤ 3 mm	1.00	1.00	1.00
	4+ mm	1.24 (0.37, 4.16)	0.76 (0.15, 3.77)	0.82 (0.16, 4.18)
Roccuzzo score	0	1.00	1.00	1.00
	1–10	1.33 (0.52, 3.39)	3.37 (1.19, 9.59)	2.79 (0.91, 8.53)
	11–24	6.20 (1.64, 23.5)	2.49 (0.34, 18.12)	1.88 (0.24, 14.60)
	25+	17.92 (4.61, 69.6)	24.76 (2.07, 296.71)	27.84 (2.11, 366.97)
Stage	1	1.00		1.00
	2	0.00		0.00
	3	1.40 (0.09, 22.36)		3.68 (0.10, 130.1)
	4	3.89 (0.52, 29.02)		10.10 (0.49, 208.2)
Grade	A	1.00		1.00
	B	1.16 (0.23, 5.98)		0.25 (0.02, 2.63)
	C	2.08 (0.48, 9.03)		0.27 (0.03, 2.56)

*Note:* Crude values are univariate. aHR_1_ adjusts for sex, age, maintenance, arch, smoking, implant/grafting parameters, keratinized mucosa, adjacent PD, and Roccuzzo score. aHR_2_ further adjusts for periodontitis stage/grade (2017 WW).

Abbreviations: HR, hazard ratio; PD, probing depth.

^a^ Side level.

### 3.7 Treatment Outcomes for PID

The therapeutic response to PID was variable. All 99 implants diagnosed with PID underwent deep cleaning; however, only 23 (23.23%) regained a healthy status. Among the 76 implants that remained unresolved, 34 underwent flap surgery with or without guided bone regeneration (GBR). Of these, 21 implants (61.76%) showed recovery. Overall, 44 (44.44%) of the 99 implants with PID returned to a clinically healthy status after treatment.

## 4 Discussion

This 28‐year retrospective cohort study provides long‐term evidence on the clinical performance of tissue‐level implants in a Taiwanese periodontal practice. At the implant level, the cumulative success and survival rates were 71.00% and 92.58%, respectively, while at the patient level, they were 38.02% and 81.38%. These findings are broadly consistent with long‐term international data, reinforcing the reliability of tissue‐level implants under well‐controlled periodontal conditions.

### 4.1 Comparison With Previous Literature

The 10‐ and 15‐year success rates, at implant level, in this study (81.01% and 74.91%, respectively) fell between those reported by Ferrigno (92.7%) [11] and Simonis (52.0%–61.0%) [8]. Similarly, our survival rates at 10, 15, and 20 years (96.86%, 94.23%, and 92.58%) compared favorably with studies by Ferrigno (95.9%) and Roccuzzo (93.0%) [13], exceeded those by Simonis (82.9% at 16 years). Notably, this study provides one of the longest follow‐up durations globally and the longest from the Asia‐Pacific region, adding novel geographical and temporal perspectives to the body of implantology evidence.

### 4.2 Enhanced Diagnostic Sensitivity for PID

A distinctive methodological aspect of this study was the use of PD ≥ 5 mm as the sole diagnostic criterion for PID, in contrast to the more common triad of PD ≧ 6 mm, bleeding/suppuration on probing (BOP/SOP), and radiographic bone loss [18, 22, 24]. This approach prioritizes diagnostic sensitivity, especially for early‐stage PID, and addresses the difficulty in detecting buccal/lingual bone loss on radiography [19, 20]. While this may slightly increase the risk of overdiagnosis, it enhances early detection and may better guide preventive interventions.

### 4.3 Risk Factor Insights

Our findings affirm well‐established risk factors such as male and severe periodontitis for both PID and loss [5, 6, 7]. Interestingly, while smoking was a significant factor in univariate analysis, it did not retain significance in multivariable models. This attenuation may be due to patient selection bias, as heavy smokers were excluded from treatment candidacy, a reflection of prudent clinical practice but a potential confounder for generalizability.

Implant length emerged as a consistent protective factor for implant survival, corroborating previous reports [1, 10, 11]. Increasing maintenance frequency was protective for implant survival.

Of note, the presence of AM (non‐KM) appeared protective for implant health. The literature on KM remains mixed: while some studies emphasize its importance for peri‐implant health [3, 36] particularly after free gingival grafting [37], others report no significant association [38]. The side‐level analysis of KM in this study, accounting for buccal vs. lingual variation, offers a nuanced contribution to this debate.

This study corroborated a similar Italian 20‐year study of a periodontal practice [26] and a series of 2024 AO/AAP consensus reports [39, 40, 41] that good control of periodontal conditions and dental biofilm, regular maintenance, and tissue‐level implants with transmucosal abutments are protective factors for long‐term success. However, periodontal disease by Roccuzzo's index showed a significant risk factor but that by 2017 WW classification did not. This could be due to the fact that Roccuzzo's index is based on all‐sites residual pockets that reflect active periodontitis whereas the 2017 WW classification is primarily based on interdental CAL or bone loss that includes history of periodontitis. Further studies are needed to elucidate this contradiction.

### 4.4 Clinical Management of PID

Despite early detection through probing, the treatment response for PID remained variable and suboptimal. Only 44.44% of implants with PID returned to a healthy status, even with a combination of non‐surgical debridement and surgical interventions. Rough fixture surface and threads make thorough removal of attached biofilms difficult. This could be the main reason for the unpredictable response of PID treatment. In addition, our experience appears that early detection and early intervention have better results of PID treatment. This is in agreement with the suggestion of American Academy of Periodontology [22]. However, further research is needed. These echo the global consensus that PID remains difficult to treat once established [16, 17, 39], underscoring the importance of prevention, early diagnosis, and regular monitoring.

### 4.5 Study Strengths

This study possesses several notable strengths that enhance the reliability and relevance of its findings. First, it offers one of the longest follow‐up durations in implant dentistry to date, with 28 years of cumulative data, thereby contributing rare insights into the ultra‐long‐term performance of tissue‐level implants. Second, the study's methodological consistency—wherein all surgical procedures, follow‐ups, and maintenance were conducted by a single board‐certified periodontist—ensured high internal validity by minimizing inter‐operator variability. Third, the inclusion of patients with stable periodontal conditions and high compliance allowed for clearer attribution of outcomes to implant‐ and procedure‐related factors, rather than systemic or behavioral confounders. Fourth, the use of a simplified and sensitive diagnostic approach—PD ≥ 5 mm as a standalone criterion for PID—enhanced the early detection and may serve as a pragmatic model for clinical practice. Lastly, the study fills a significant gap in the literature by presenting long‐term implant outcomes from an Asian cohort, broadening the geographic scope of evidence in a field historically dominated by Western populations.

### 4.6 Limitations

The study's strengths also entail certain limitations. Using PD as the sole criterion for PID relies on probing which has inherent technique error [42]. A single experienced examiner throughout the study may have minimized the error. But the single‐clinician, single‐center setting may limit the generalizability of findings to other populations and clinical workflows, particularly general practices. Moreover, only one implant system was evaluated, preventing comparisons across different brands or platform types. Finally, the exclusion of high‐risk individuals (e.g., heavy smokers or medically compromised patients) [39] restricts the applicability of findings to more complex patient profiles.

### 4.7 Clinical Implications and Future Directions

This study suggests that long‐term implant success and survival are achievable under strict periodontal maintenance and careful case selection. The use of PD ≥ 5 mm as a standalone screening tool could facilitate earlier detection and intervention for peri‐implant disease, especially in clinical settings with limited access to CBCT.

Future research should validate this diagnostic simplification in broader and more diverse populations, and explore adjunctive biomolecular or imaging markers to enhance specificity. Comparative studies between different implant types, surface technologies, and patient comorbidities are also warranted.

## 5 Conclusion

Tissue‐level implants placed and maintained in a specialized periodontal setting demonstrated excellent long‐term success and survival rates over a 28‐year period. The study supports the value of rigorous periodontal care, early detection strategies, and regular maintenance in optimizing long‐term implant outcomes. Simplifying diagnostic criteria to focus on probing depth may provide a practical, sensitive approach for identifying peri‐implant disease in routine practice.

## Author Contributions

**Hongmin Lai:** conceptualization, investigation, writing – original draft, data curation, writing – review and editing, supervision. **Ming‐Tsuen Lin:** writing – review and editing. **Ting‐Yu Lin:** writing – review and editing, formal analysis. **Sam Li‐Sheng Chen:** formal analysis, writing – review and editing. **Tony Hsiu‐Hsi Chen:** writing – review and editing, supervision, validation. **Amy Ming‐Fang Yen:** formal analysis, writing – review and editing, data curation, methodology.

## Funding

This study was supported by the Ministry of Science and Technology, Taiwan (113‐2314‐B‐038‐085‐MY3). The funders had no role in the design or conduct of the study; collection, management, analysis, or interpretation of the data; preparation, review, or approval of the manuscript; or decision to submit the manuscript for publication.

## Conflicts of Interest

The authors declare no conflicts of interest.

## Data Availability

The data that support the findings of this study are not publicly available.
